# Recurrent acute myocardial infarction with coronary artery aneurysm in a patient with Behçet's disease: a case report

**DOI:** 10.4076/1752-1947-3-8869

**Published:** 2009-08-20

**Authors:** Sepideh Sokhanvar, Marjaneh Karimi, Abdolreza Esmaeil-zadeh

**Affiliations:** 1 Cardiology Department, Medical Science University, Metabolic Research Center, Zanjan, Iran; 2 Rheumatology Department, Medical Science University, Metabolic Research Center, Zanjan, Iran; 3 Immunology Department, Medical Science University, Metabolic Research Center, Zanjan, Iran

## Abstract

**Introduction:**

Behçet's disease is an inflammatory disorder of unknown origin, with mucocutaneous, ocular, articular, vascular, gastrointestinal and central nervous system manifestations. Although cardiac involvement is not an uncommon manifestation of Behçet's disease, coronary aneurysm has rarely been reported.

**Case presentation:**

A 36-year-old Iranian man was admitted to our emergency department for retrosternal pain of two and a half hours duration. His detailed medical history revealed that he had no risk factors for coronary artery disease, however, Behçet's disease had been diagnosed about 10 years earlier. His electrocardiogram showed inferior myocardial infarction. He underwent coronary angiography that showed multiple giant aneurysms in his coronary arteries. Two months later, he experienced another episode of unstable angina. This was followed by two episodes of anterior myocardial infarction 2 and 5 months afterwards.

**Conclusion:**

This case highlights the importance of careful diagnostic work-up in the evaluation of myocardial infarction in patients. In our patient, Behçet's disease proved to be the cause of recurrent myocardial infarction.

## Introduction

Coronary artery disease is one of the most common causes of acute myocardial infarction. In addition to atherosclerosis, coronary vasculitis may also cause acute coronary syndrome [1]-[3]. One of the most common characteristics of Behçet's disease (BD) is vasculitis, which rarely involves the coronary arteries [4,5]. However, data on the clinical significance of coronary artery involvement are very limited. In BD, acute myocardial infarction is a serious complication and an important clinical manifestation of coronary arteritis. Myocardial infarction due to coronary vasculitis in BD is infrequent and few cases have been documented in the literature [1]-[3]. We report the case of a patient with BD presenting with recurrent myocardial infarction due to coronary vasculitis with diffuse fusiform coronary aneurysms.

## Case presentation

A 36-year-old Iranian man was admitted to our emergency department on 31 August 2006, presenting with epigastric and retrosternal pain of two and a half hours duration. His detailed medical history revealed that he had no risk factors for coronary artery disease, however, BD had been diagnosed 10 years earlier and he was on colchicine 1 mg/day. Oral aphthous ulcers were exacerbated during coronary artery events. Eye examination was normal. He had an episode of thrombophlebitis of his left leg in 2004 and had been taking warfarin but had discontinued the medication.

Medical examination revealed blood pressure of 130/70 mmHg and heart rate of 85 beats/minute and the patient was pale and perspiring. Chest auscultation revealed no abnormalities. His electrocardiogram (ECG) revealed normal sinus rhythm with ST segment elevation on II, III and aVF and reciprocal ST segment depression on V1-V6. Laboratory tests showed elevation of plasma total creatine phosphokinase (CPK) and CPK-MB activities. There were no findings consistent with coagulation and fibrinolysis disorders. He was diagnosed as having an acute inferior wall myocardial infarction, therefore nitroglycerin, heparin, aspirin and beta blocker therapies were started immediately followed by thrombolytic therapy with streptokinase within 1 hour. After 40 minutes, his chest pain was relieved and there was a significant reduction of ST segment elevation along with increased cardiac enzyme levels. A transthoracic echocardiogram showed mild apical hypokinesia and a left ventricular ejection fraction of 50%.

**Figure 1:**
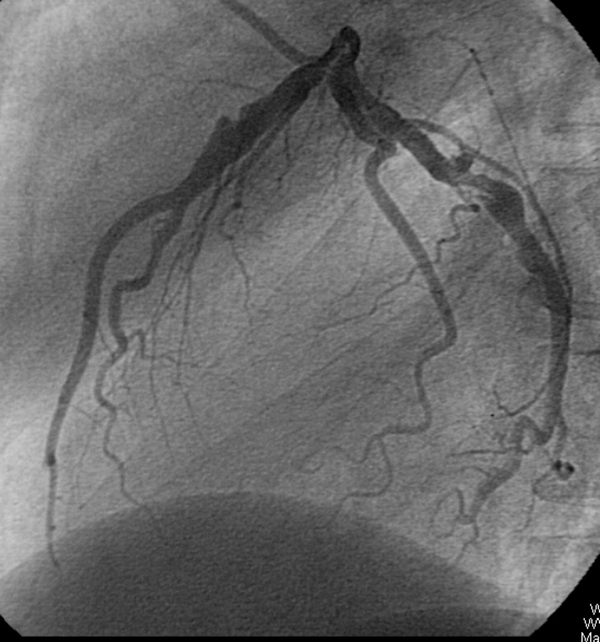
**Coronary angiogram after left coronary administration of contrast material in the left anterior oblique position**. The angiogram revealed an 8 mm giant aneurysm of the proximal left anterior descending artery and an 8 mm aneurysm of the proximal left circumflex coronary artery.

**Figure 2:**
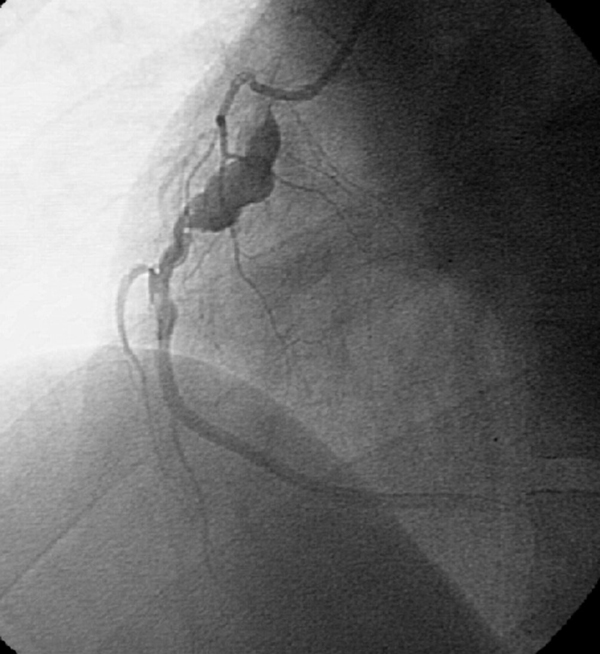
**Coronary angiogram after right coronary administration of contrast material in the left anterior oblique position**. The angiogram revealed a 9 mm aneurysm of the proximal right coronary artery.

On the third day of hospital admission, he underwent coronary angiography. Coronary angiography revealed an 8 mm giant aneurysm of the proximal left anterior descending artery, an 8 mm aneurysm of the proximal left circumflex coronary artery (Figure 1) and a 9 mm aneurysm of the proximal right coronary artery (Figure 2). Based on clinical evidence, electrocardiogram and coronary angiography, we considered that the acute myocardial infarction in our patient was due to a coronary aneurysm. Therefore, we did not attempt any coronary intervention and decided to continue with medical therapy including azathioprine, colchicine, prednisolone, aspirin, beta blocker, nitroglycerin and enalapril. On 25 November 2006, the patient experienced another episode of chest retrosternal pain which lasted for 7 hours. His ECG revealed significant ST segment depression on V1-V6. Cardiac enzymes did not rise so he was diagnosed with unstable angina. No changes in ECG were noticed after 5 days. The patient was not adhering to drug therapy and when his coronary events occurred drug therapy was restarted. The next episode of retrosternal pain occurred on 28 February 2007 and lasted for 5 hours. His ECG showed tall T waves on precordial leads. Laboratory tests showed elevation of plasma total CK and CK-MB activities. He was diagnosed with an acute anterior wall myocardial infarction. Medical treatment was started but since he had received thrombolytic therapy 5 months earlier, streptokinase was not administered. The echocardiogram showed septal, apical and anterior wall hypokinesia with an estimated left ventricular ejection fraction of about 25-30%. He was discharged 10 days later with his previous medication plus digoxin, warfarin, frusemide, and spironolactone. On 2 August 2007, he experienced another bout of retrosternal pain that lasted for 7 hours. ECG showed ST segment elevation on precordial leads. Laboratory tests showed elevation of plasma total CK and CK-MB activities. This time, a new anterior myocardial infarction was diagnosed, medical treatment was started and azathioprine was switched for pulse cyclophosphamide 10 mg/kg, but the patient then discharged himself. At the time of writing, the patient is well with pulse cyclophosphamide every 2 months and prednisolone 7.5 mg/day, but unfortunately, he is non-compliant to drug therapy and so it was not possible to switch him to oral medication.

## Discussion

Vascular involvement is a well-known characteristic of BD. Large vessel disease or acute cerebrovascular infarction in the setting of aphthosis should suggest BD [6]. It can occur even in relatively young patients with no vascular risk factors. Arterial involvement is generally expressed by thrombosis, stenosis and/or aneurysms, as shown in early studies [1]-[5]. The prevalence of coronary involvement in BD is 0.5% [7]. Aneurysms are seen less frequently than arterial occlusions [8]. The pathogenesis of the arterial lesion in BD has been well documented. An inflammatory obliterative endarteritis of the vasa vasorum, most likely brought about by immune deposition, causes destruction of media and fibrosis and thus weakens and predisposes the arterial wall to aneurysm formation that eventually ruptures [9]. In our patient, angiography was performed for evaluation of coronary anatomy. Coronary angiograms revealed multiple giant aneurysms of all coronary arteries. There was no evidence of underlying disease such as antiphospholipid syndrome, rheumatic or connective tissue disorders. In the literature, several different therapeutic approaches have been advanced for the management of myocardial infarction in patients with BD. These may include primary percutaneous transluminal coronary angioplasty (PTCA) [10,11], repair of the aneurysm [12], or administration of fibrinolytic therapy at the early stages of acute myocardial infarction, as in our patient. Finally, the therapeutic approach remains complex, especially in recurrent myocardial infarction. It is important to perform surgical and/or interventional radiological procedures to intervene urgently for enlarging ischaemia, ruptured aneurysms or organ-threatening ischaemia. However, unless intervention is urgently required, it is prudent to postpone invasive approaches in these vascular complications until the inflammatory component of BD has been controlled with medical therapy [13].

## Conclusion

For patients with acute myocardial infarction without any other risk factor and especially in relatively young patients, Behçet's disease should be considered as the underlying pathology.

## Consent

Written informed consent was obtained from the patient for publication of this case report and any accompanying images. A copy of the written consent is available for review by the Editor-in-Chief of this journal.

## Competing interests

The authors declare that they have no competing interests.

## Authors' contributions

SS performed the coronary angiography and was a major contributor in writing the manuscript. MK and AEZ were involved in the diagnostic work-up and management of Behçet's disease in our patient. All authors read and approved the final manuscript.

## References

[B1] Ioakimidis D Georganas C Panagoulis C Gournizakis A Iliopoulos A Kremastinos D Kontomerkos T A case of Adamantiadis-Behcet's syndrome presenting as myocardial infarction Clin Exp Rheumatol 1993 11 183 186 8508561

[B2] Kawakami Y Nakayama Y Nagao H Hirota Y Kawamura K A case of Behcet's disease complicated with acute myocardial infarction Kokyu To Junkan 1991 39 935 938 [Japanese] 1749873

[B3] Le Thi HD Wechsler B Kahn JC Benhamou E Cajfinger F Le HP Godeau P Myocardial infarction in Behcet's disease Arch Mal Coeur Vaiss 1987 80 1663 1667 3128210

[B4] Bowles CA Nelson AM Hammill SC O'Duffy JD Cardiac involvement in Behcet's disease Arthritis Rheum 1985 28 345 348 10.1002/art.1780280317 3977978

[B5] Wechsler B Du LT Kieffer E Cardiovascular manifestations of Behcet's disease Ann Med Interne (Paris) 1999 150 542 554 10637670

[B6] Silman A Gul A Is there a place for large vessel disease in the diagnostic criteria of Behcet's disease? J Rheumatol 2000 27 2050 2051 10955355

[B7] Hutchison SJ Belch JJ Behcet's syndrome presenting as myocardial infarction with impaired blood fibrinolysis Br Heart J 1984 52 686 687 10.1136/hrt.52.6.686 6508969 PMC481707

[B8] Kosar F Sahin I Gullu H Cehreli S Acute myocardial infarction with normal coronary arteries in a young man with the Behcet's disease Int J Cardiol 2005 99 355 357 10.1016/j.ijcard.2003.11.039 15749205

[B9] Matsumoto T Uekusa T Fukuda Y Vasculo-Behcet's disease: a pathologic study of eight cases Hum Pathol 1991 22 45 51 10.1016/0046-8177(91)90060-3 1985077

[B10] Drobinski G Wechsler B Pavie A Artigou JY Marek P Godeau P Grosgogeat Y Emergency percutaneous coronary dilatation for acute myocardial infarction in Behcet's disease Eur Heart J 1987 8 1133 1136 2960524 10.1093/oxfordjournals.eurheartj.a062179

[B11] Tamura Y Matsuoka A Ohtaki E Okabe M Shibata A Behcet's disease complicated by acute myocardial infarction treated with percutaneous transluminal coronary angioplasty Kokyu To Junkan 1988 36 341 346 [Japanese] 2967530

[B12] Ozeren M Dogan OV Dogan S Yucel E True and pseudo aneurysms of coronary arteries in a patient with Behcet's disease Eur J Cardiothorac Surg 2004 25 465 467 10.1016/j.ejcts.2003.11.040 15019683

[B13] Calamia KT Schirmer M Melikoglu M Major vessel involvement in Behcet disease Curr Opin Rheumatol 2005 17 1 8 10.1097/01.bor.0000145520.76348.dd 15604898

